# Introduction of Tropical Plants into Florida

**Published:** 1833

**Authors:** 


### Art. XVI. Introduction of Tropical Plants into Florida.

Dr. Perrine, for several years our Consul in the Province of Yucatan, has been making zealous efforts to naturalize a variety of tropical plants in West Florida; and has memorialized Congress on the subject of a grant of lands for that purpose. In reference to our national resources, and, especially, to our Materia Medica, the object is one of considerable interest, and ought to be encouraged by the profession. We trust that those who may think with us on this matter, will be willing to exert whatever influence they may possess over their representatives in Congress, for the purpose of furthering this important experiment, which must necessarily be attended with many difficulties and sacrifices.

